# Durability of Basalt- and Glass Fiber-Reinforced Polymers: Influence of Internal Stresses, Mass Loss Modeling, and Mechanical/Thermomechanical Properties Under Extreme Cold Climate Exposure

**DOI:** 10.3390/polym17182457

**Published:** 2025-09-11

**Authors:** Anatoly K. Kychkin, Oleg V. Startsev, Mikhail P. Lebedev, Anatoly S. Krotov, Aisen A. Kychkin, Anna A. Gavrilieva

**Affiliations:** 1 V.P. Larionov Institute of the Physical-Technical Problems of the North of the Siberian Branch of the RAS, 1, Oktyabrskaya Str., Yakutsk 677980, Russia; startsevov@gmail.com (O.V.S.); askrotov@list.ru (A.S.K.); gav-ann@yandex.ru (A.A.G.); 2 Federal Research Centre “The Yakut Scientific Centre of the Siberian Branch of the Russian Academy of Sciences”, 2, Petrovskogo Str., Yakutsk 677000, Russia; m.p.lebedev@mail.ru (M.P.L.); icen.kychkin@mail.ru (A.A.K.)

**Keywords:** BFRP, GFRP, laminate, extreme cold climate, outdoor exposure, degradation, tensile strength, glass transition temperature (Tg), coefficient of linear thermal expansion (CLTE)

## Abstract

The durability of basalt fiber-reinforced polymer (BFRP) and glass fiber-reinforced polymer (GFRP) composites was evaluated under extreme cold conditions in Yakutsk (−54 to +36 °C. Laminates (18 layers, epoxy CYD-128) were exposed outdoors for three years. Mechanical testing showed tensile strength and modulus reductions of 22–32% for GFRP, compared with only 6–12% for BFRP. Dynamic mechanical analysis indicated that the glass transition temperature decreased by 11–14 °C in GFRP and 4–6 °C in BFRP. Mass loss kinetics were studied on specimens of different sizes (10 × 10, 20 × 20, and 40 × 40 mm) over 405 days. Seasonal sorption ranged between 0.01–0.19%, while long-term degradation followed a Fickian law with diffusion coefficients of degradation products from $1\times 10^{-4}$ to $0.29\;{\mathrm{mm}}^{2}/\mathrm{day}$. A diffusion-based model was proposed, where total mass change is represented as the superposition of reversible sorption and irreversible degradation. The model accurately reproduced experimental trends, highlighting the higher resistance of BFRP. Surface morphology analysis revealed matrix erosion and microcracking on exposed surfaces, with average roughness increasing from 1.61–5.61 µm to 5.86–11.73 µm. Thermomechanical analysis confirmed that BFRP maintained more stable coefficients of linear thermal expansion (−60 to 100 °C) than GFRP, reducing thermally induced stresses during seasonal cycles. These findings demonstrate the superior stability of BFRP compared with GFRP under cold-climate exposure. Comparison of experimental results with mathematical modeling demonstrated that the primary cause of polymer matrix degradation is the action of abrupt internal stresses arising during thermal cycling under extreme cold climate conditions.

## 1. Introduction

Basalt fiber reinforced polymers (BFRP), manufactured in the form of unidirectional rods or fabric laminates, have attracted attention due to the combination of high strength, low density, and corrosion resistance. These properties make them competitive with traditional glass fiber reinforced polymers (GFRP) and in demand in the aerospace, shipbuilding, automotive, and energy industries [1,2,3,4]. An important advantage of basalt fibers is their minimal environmental impact compared with carbon, glass, and other mineral fibers [1]. After chemical surface treatment, the fibers demonstrate improved adhesion to the polymer matrix, which positively affects the strength and durability of the composites. The application of BFRP in structures subjected to dynamic loads, as well as in the reinforcement of concrete and masonry structures, was discussed in the studies of Elmahdy and Verleysen [2], Duan et al. [3], and Yan et al. [5], who showed an increase in load-bearing capacity of up to 60% and a service life extension of up to 50 years. The prospects of BFRP in civil engineering, infrastructure projects, and power transmission lines were confirmed by Liu et al. [6] and Monaldo et al. [7]. Basalt fiber-reinforced polymer (BFRP), offering advantages such as lower cost and superior creep resistance over traditional composites, is attracting increasing attention in construction, making its comprehensive study of properties, applications, and challenges highly relevant [3]. In addition to high strength, basalt fiber-based materials also exhibit improved insulation properties and bending resistance [4].

To assess the durability of BFRP in various aggressive environments, comparative experiments with GFRP were carried out [8,9,10,11,12,13,14]. Wang Z. et al. [8,9] and Wang M. et al. [10] showed that BFRP had a more stable interface with the epoxy matrix and lower sensitivity to alkaline attack compared with GFRP. Wu et al. [11], Hashim et al. [12], and Lukachevskai et al. [14] demonstrated high resistance of BFRP to water, salt, and ultraviolet (UV) corrosion, whereas GFRP degraded more severely. Zhao et al. [13] described in detail the mechanisms of photo-aging, including the loss of adhesion at the polymer–filler interface. BFRP also shows higher resistance to fatigue loads [15], erosion [16], and low-velocity impacts [17].

The aging of BFRP under natural conditions was analyzed by Startsev et al. [18], who identified the influence of temperature, thermal cycling, humidity, and UV radiation on mechanical properties. In cold climates, low temperatures generated significant internal stresses due to the mismatch of thermal expansion coefficients between the polymer matrix and reinforcing fibers [18,19,20,21,22], which promoted the formation of microcracks and macro-damage, especially in the presence of moisture [23,24]. Internal stresses in the matrix can be estimated by the equation

(1) $$\sigma_{m}=-E_{m}\alpha_{m}\left(T-T_{0}\right),$$

where $\sigma_{m}$ is the tensile internal stress in the matrix, $E_{m}$ is the elastic modulus of the matrix, $\alpha_{m}$ is the coefficient of linear thermal expansion of the matrix at the measurement temperature *T*, and $T_{0}$ is the curing temperature of the matrix. For GFRP and BFRP investigated by Lukachevskai et al. [14], at the winter temperature in Yakutsk of −40 °C, the values of $\sigma_{m}$ reach 35±5MPa. Changes in the mass of exposed specimens were used to evaluate degradation, accounting for both moisture sorption and polymer matrix damage [25,26,27,28], while dynamic mechanical analysis (DMA) and thermomechanical analysis (TMA) provided information on glass transition, segmental mobility, and microstructural changes [24,29,30,31,32,33,34,35].

Despite the large number of studies on the properties of BFRP and GFRP in various environments, the literature lacks comprehensive data on the behavior of these composites under cold climate conditions that simultaneously integrate mechanical degradation, diffusion-based modeling sorption–desorption mass changes and changes in the state of the epoxy matrix.

TTherefore, the present study aims to conduct a comparative investigation of BFRP and GFRP under extreme cold climate conditions, focusing on changes in mechanical properties, binder content, surface degradation (surface morphology and roughness), mass loss in hangar and on open stands (both in model and experimental setups), and the state of the epoxy matrix (DMA, TMA). An integrated approach is proposed to evaluate the influence of internal stresses on the durability of BFRP and GFRP in cold climates.

## 2. Materials and Methods

### 2.1. Materials

For the purpose of the study, specimens in the form of GFRP and BFRP plates were fabricated. As reinforcing fillers, glass fabric grade TR-560-30A (manufactured by JSC “Polotsk-Steklovolokno”, Polotsk, Belarus) and basalt fabric grade BT-11 (100) (manufactured by LLC “Technical Fabrics Factory”, Vladimir, Russia) were used. The main physical and mechanical properties of the fabrics are summarized in Table 1.

**Scheme 1:**
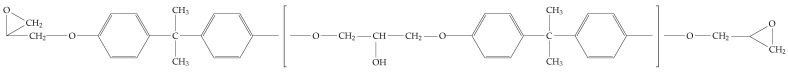
The chemical structure of the diglycidyl ether of bisphenol A (CYD-128).

**Scheme 2:**
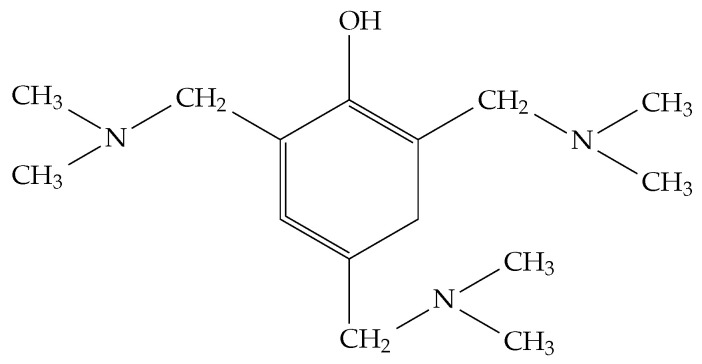
The chemical structure of the isomethyltetrahydrophthalic anhydride (iso MTHPA).

**Scheme 3:**
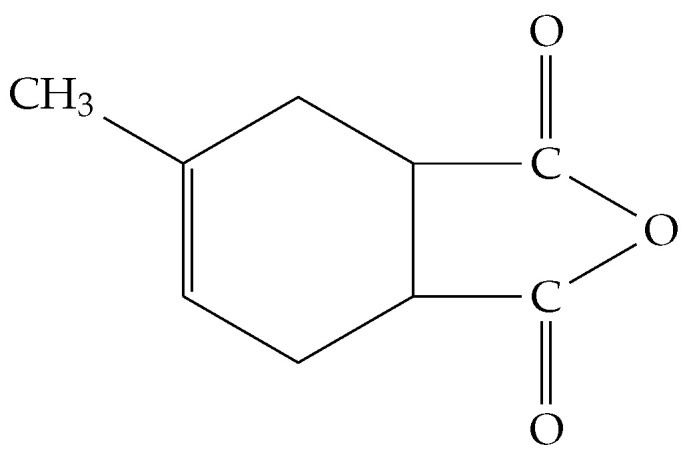
The chemical structure of the 2,4,6-tris(dimethylaminomethyl)phenol (DMP-30).

The binder matrix was based on epoxy resin CYD-128 (Hefei TNJ Chemical Industry Co., Ltd., Hefei, China; Scheme 1) [36], cured with iso-methyltetrahydrophthalic anhydride (iso-MTHPA; Scheme 2) , in the presence of the accelerator 2,4,6-tris(dimethylaminomethyl)phenol UP-606/2 (Sterlitamak Petrochemical Plant JSC, Russia; Scheme 3). The epoxy resin CYD-128 was chosen as the matrix material because it is a domestic analog of ED-22, meeting all technical specifications including viscosity, curing time, and mechanical properties after curing. The choice of CYD-128 also ensures continuity with our previous studies on ED-20 and ED-22 resins. This resin possesses the necessary processing characteristics for use with basalt and glass fibers and exhibits high mechanical performance after curing. The detailed characteristics of the GFRP and BFRP plates are presented in our previous study [14].

**Figure 1:**
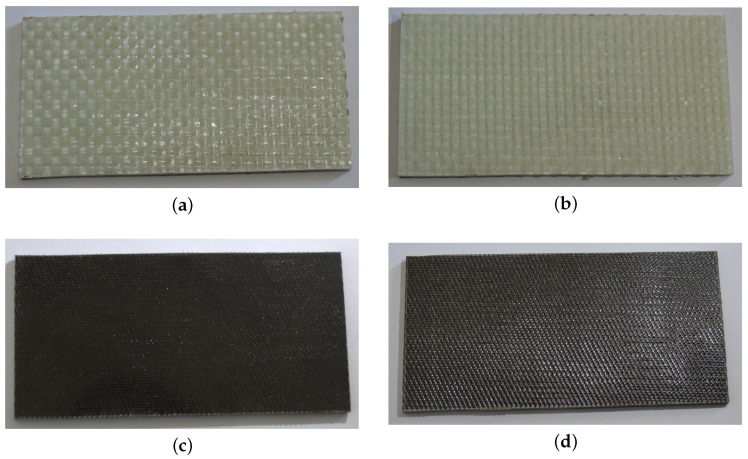
Photographs of the fabricated samples: (**a**) a mold side of GFRP, (**b**) a bag side GFRP, (**c**) a mold side of BFRP, (**d**) a bag side BFRP.

GFRP plates (14 layers) and BFRP plates (18 layers) were manufactured using the vacuum infusion method, followed by curing in molds at 160±2 °C for 4 h. The fabricated plates had dimensions of 950×450×5.2±0.1mm. The fabricated samples are shown in Figure 1. The epoxy matrix content was 22.9 ± 0.04 wt% in GFRP and 28.1 ± 0.04 wt% in BFRP.

### 2.2. Material Exposure

GFRP and BFRP plates were exposed with the mold side facing the sun for three years in Yakutsk on outdoor racks (54 cm above ground, $45^{\circ }$ inclination). Properties were evaluated in the as-received (initial) state and after 1, 2, and 3 years of exposure, corresponding to the initial phase of long-term degradation studies. Further investigations are planned for 5- and 10-year exposure periods to evaluate later stages of material aging.

### 2.3. Mass Change Measurement

Two additional specimen sets (10 × 10, 20 × 20, 40 × 40 mm; four replicates per size) were dried, measured, and weighed prior to exposure. One set was placed outdoors (19 April 2024–30 May 2025; 405 days), the other in an unheated indoor shed simulating ambient temperature without solar UV. Thickness and mass were recorded 193 times (∼every 2 days) using a micrometer (±0.01 mm) and electronic balance (±0.0001 g).

### 2.4. Matrix Content

Epoxy content was determined by calcining specimens at 600 °C for 6 h in porcelain crucibles, followed by weighing on an analytical balance (±0.0001 g). Mass fraction was calculated from the difference between initial and post-calcination mass.

### 2.5. SEM and Surface Roughness

Surface degradation and microstructural changes were examined using a scanning electron microscope (SEM, JSM-7800F, JEOL, Tokyo, Japan) operated at low accelerating voltage under high-vacuum conditions. The arithmetic average surface roughness (Ra, µm) was determined using a portable profilometer Surftest SJ-201P (Mitutoyo, Kawasaki-shi, Japan). Following GOST 2789-73 [37], surface roughness was evaluated using the arithmetic mean deviation of the profile (Ra), which is considered the most representative parameter. The $R_{a}$ parameter was calculated as the arithmetic mean of the absolute deviations of the surface profile ($Y_{i}$) from the mean line over the evaluation length (*l*):

(2) $$R_{a}=\frac{1}{n}\sum_{i=1}^{n}\left|Y_{i}\right|,$$

### 2.6. Tensile Testing

Tensile strength ($\sigma_{t}$) and modulus ($E_{t}$) were determined per ASTM D3039/ D3039M-17 [38] using five specimens per composite type (10 × 250 ± 2 mm) with 10 × 75 ± 2 mm tabs inclined at $30^{\circ }$. Gauge length was ≥95 ± 2 mm, test speed 2 mm/min, extensometer gauge length 50 mm. Specimens were dried at 60 °C for 72 h prior to testing.

### 2.7. Dynamic Mechanical Analysis (DMA)

DMA was performed per ISO 6721 [39] using a DMA 242C (Netzsch, Selb, Germany) in three-point bending (span 40 mm), 25–150 °C, 5 °C/min, 10 µm amplitude, 1 Hz, under argon. Glass transition temperature ($T_{g}$) and transition limits ($T_{g1}$, $T_{g2}$) were determined from $E^{\prime }\left(T\right)$ inflection points or $E^{\prime \prime }$ maxima. Face and back sides were analyzed separately.

### 2.8. Thermomechanical Analysis (TMA)

CLTE was measured per ISO 11359-2 [40] on a TMA 202 C (Netzsch) at 5 °C/min in helium (70 ml/min), 3 cN load [18]. Thickness change $\Delta H=H-H_{0}$, relative change $\Delta H/H_{0}$, and CLTE

(3) $$\alpha =\frac{1}{H}\frac{\partial H}{\partial T}$$

were determined perpendicular to the reinforcement plane from −60 to 100 °C, where *H* is thickness at temperature *T* and $H_{0}$ is initial thickness.

## 3. Results and Discussion

### 3.1. Climate Conditions of Exposure

**Figure 2:**
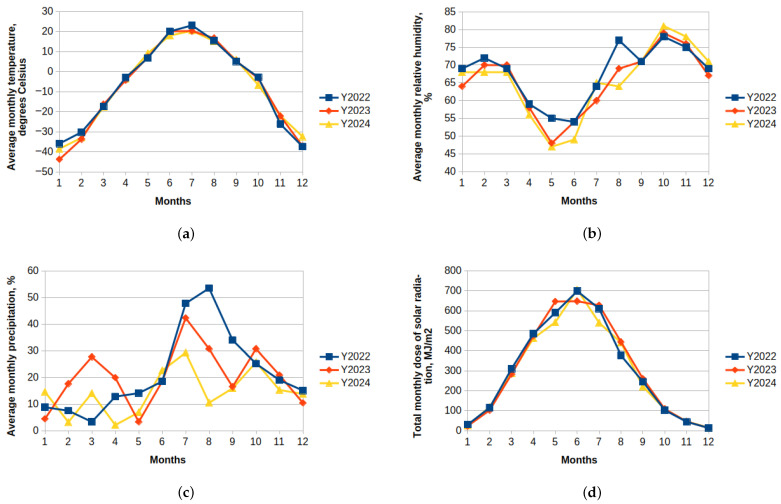
Average monthly climatic parameters in Yakutsk for the three-year exposure period of GFRP and BFRP samples: (**a**) air temperature, (**b**) relative humidity, (**c**) total solar radiation dose, (**d**) precipitation.

Figure 2 shows the average monthly climatic parameters of Yakutsk over the three-year exposure period of glass fiber reinforced polymer (GFRP) and basalt fiber reinforced polymer (BFRP) samples (2022–2024).

The climate of Yakutsk is characterized by a sharply continental type with low precipitation (about 230 mm/year) and an average relative humidity of approximately 68%. The main feature is the large temperature amplitude, with a difference between winter and summer temperatures reaching about 90 °C. The winter period lasts from October to April, while spring and autumn are very short. Internal stresses in polymer composites increase notably during these transition seasons and depend on the relationship Equation (1).

Despite the reduced sunlight duration in winter, the annual total solar radiation dose was approximately 3600 MJ/m^2^, consistent with previously reported data [41] (3516 MJ/m^2^). Table 2 presents the cumulative number of temperature transitions for GFRP and BFRP during the 2023 and 2024 years, corresponding to extremely cold environmental conditions.

### 3.2. Changes in Mechanical Properties After Exposure

Table 3 summarizes the tensile strength $\sigma_{t}$ and tensile modulus $E_{t}$ of GFRP and BFRP samples before and after 1, 2, and 3 years of outdoor exposure in Yakutsk. Mechanical properties of the composites was measured for five specimens of each type. Results are presented as mean ± standard deviation (SD, *n* = 5).

After 1 year of exposure, the tensile strength $\sigma_{t}$ of GFRP decreased by 11%, and the modulus $E_{t}$ decreased by 21%. These reductions continued over subsequent years, reaching 24–32%. BFRP showed similar trends but with less degradation (6–12% after 3 years). These results align with previous studies [12,14,18] that report higher durability of BFRP compared to GFRP under aggressive environmental conditions.

### 3.3. Changes in Binder Mass Fraction and Surface Morphology After Exposure

Table 4 presents the binder (epoxy matrix) mass fraction determined by ashing method before and after exposure in extreme cold climate. Mass fraction of the binder in the composite (wt%) was measured for 3 specimens of each type. Results are presented as mean ± standard deviation (SD, *n* = 3).

Table 5 presents the relative binder mass fraction (wt%) defined as the ratio of the binder mass fraction after exposure to its initial value.

Reduction in binder content is related to the mechanochemical degradation of the matrix due to internal stresses [22,23,24]. A comparison of GFRP and BFRP shows that the degradation rate differs significantly. For GFRP, the binder content decreases steadily from 24.8% to 19.4% over three years, corresponding to a loss of almost 27% of the initial binder (Table 5). In contrast, BFRP demonstrates only a slight reduction almost 8% of the initial binder.

**Figure 3:**
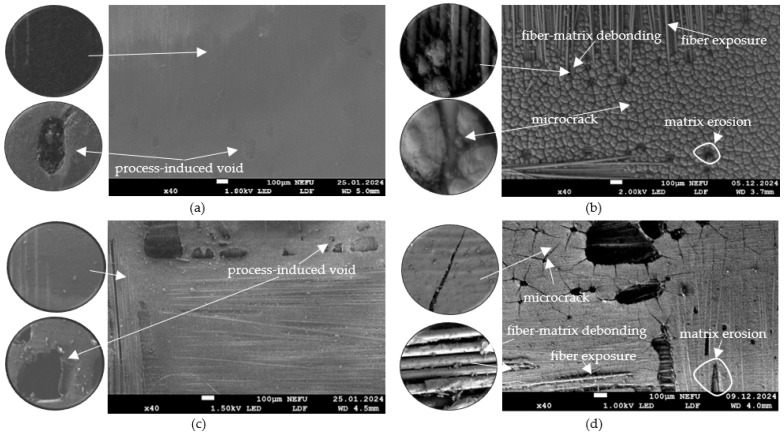
Surface morphology of GFRP under SEM: sun-facing surface (**a**) before exposure, (**b**) after 3 years; shaded surface (**c**) before exposure, (**d**) after 3 years.

**Figure 4:**
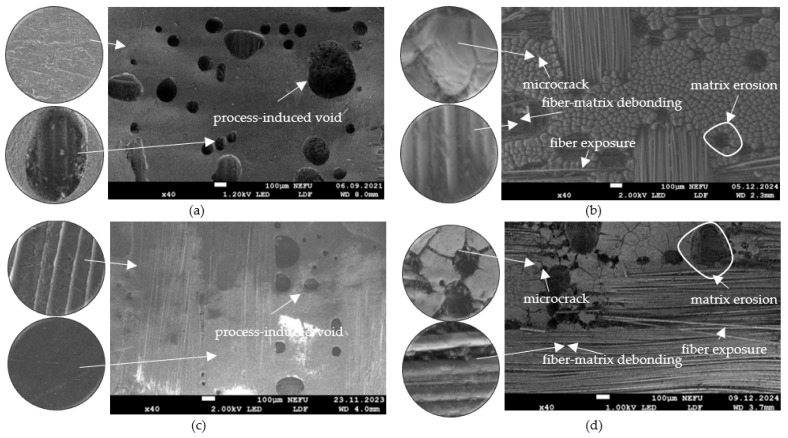
Surface morphology of BFRP under SEM: sun-facing surface (**a**) before exposure, (**b**) after 3 years; shaded surface (**c**) before exposure, (**d**) after 3 years.

The effects of environmental exposure on the surface morphology of glass- and basalt-fiber reinforced polymer composites were investigated using scanning electron microscopy (SEM). Figure 3 and Figure 4 present representative SEM images of GFRP and BFRP surfaces, respectively, highlighting differences between sun-facing and shaded sides before and after three years of outdoor exposure. Binder degradation and subsequent fiber debonding were observed, particularly on surfaces exposed to solar radiation.

**Figure 5:**
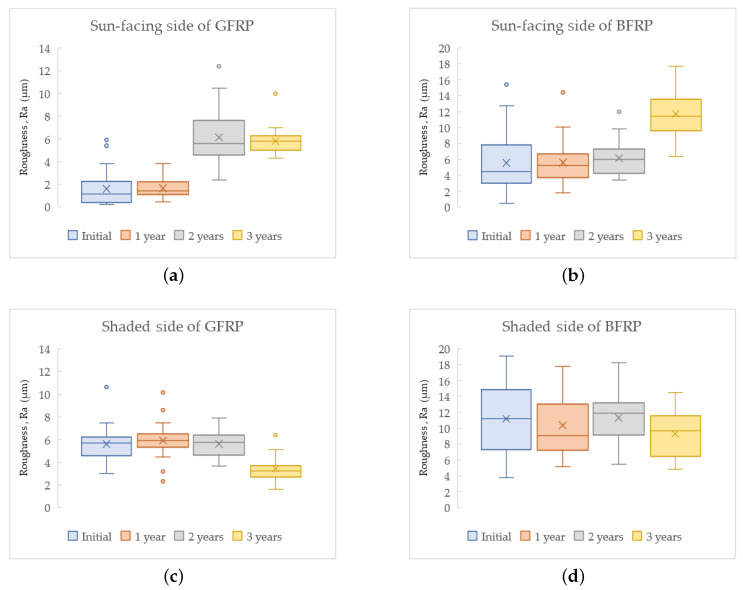
Variation in surface roughness $R_{a}$ Equation (2) over the years represented as box-plots: sun-facing surface of (**a**) GFRP, (**b**) BFRP; shaded surface of (**c**) GFRP, (**d**) BFRP.

The extent of surface damage is evidenced by the increase in the arithmetic mean roughness parameter ($R_{a}$) after open-stand exposure. Each reported value represents the mean of at least 60 measurements obtained at different surface locations to ensure reproducibility. As shown in Table 6 and Figure 5, indicating that both composites undergo surface degradation manifested as microcracking, resin erosion.

### 3.4. Mass Loss Kinetics: Model and Experiment

Prolonged exposure to climatic factors leads to changes in the mass of polymer composite materials (PCMs) such as GFRP and BFRP. Existing data [22,23,24] indicate that mass loss is associated with mechanical degradation of the polymer matrix caused by internal stresses arising from the mismatch in the coefficients of linear thermal expansion (CLTE) between the matrix and the reinforcing fibers. Additionally, UV radiation exacerbates the degradation processes. On the other hand, a seasonal mass increase with an annual periodicity, attributed to moisture sorption, has been reported in [25,26,28] for various polymer composite materials exposed to open-air climatic conditions.

#### 3.4.1. Model

The mass change of polymer composite materials exposed to extreme cold conditions is modeled as a superposition of two competing processes: moisture sorption, which increases the mass, and matrix degradation, which decreases the mass. The relative mass change at time *t*, denoted as w(t), can be expressed as

(4) $$w\left(t\right)=w_{s}\left(t\right)-w_{d}\left(t\right),$$

where $w_{s}\left(t\right)$ represents the mass change due to moisture sorption, and $w_{d}\left(t\right)$ corresponds to the mass loss due to degradation caused by solar ultraviolet (UV) radiation and internal thermal stresses.

The relative mass change is defined as

(5) $$w\left(t\right)=\frac{m_{t}-m_{0}}{m_{0}}\times 100\%,$$

where $m_{0}$ is the initial dry mass of the specimen, and $m_{t}$ is the mass of the specimen after exposure for a duration *t*.

The sorption term $w_{s}\left(t\right)$ is described by a periodic function accounting for seasonal fluctuations in moisture content [28]:

(6) $$w_{s}\left(t\right)=w_{s0}+w_{s1}\sin\left(\frac{2\pi t}{T}+\varphi \right),$$

where $w_{s0}$ is the average moisture content over the exposure period (%), $w_{s1}$ is the amplitude of seasonal moisture fluctuations (%), T=365 days is the period of seasonal variation, and φ is the phase shift (in radians).

It is assumed that low-molecular weight degradation products of the epoxy binder at the micro scale “randomly jump” through the free volume of the binder and migrate into the external environment. Furthermore, surface matrix erosion is assumed to exhibit a stochastic character. So the degradation-induced mass loss kinetics $w_{d}\left(t\right)$ is modeled by the one-dimensional solution to Fick’s diffusion second law with constant parameters [42,43], given by

(7) $$w_{d}\left(t\right)=w_{d0}\left(1-\frac{8}{\pi^{2}}\sum_{n=0}^{\infty }\frac{\exp(-\pi^{2}{\left(2n+1\right)}^{2}\frac{D}{R^{2}}t)}{{\left(2n+1\right)}^{2}}\right),$$

where an one-dimensional sample size is introduced, reflecting the dependence on the sample geometry

(8) $$\frac{1}{R^{2}}=\frac{1}{L^{2}}+\frac{1}{W^{2}}+\frac{1}{H^{2}},$$

$w_{d0}$ is the limiting mass loss due to complete degradation (%), *D* is the diffusion coefficient of degradation products (mm^2^/day), and *L*, *W*, and *H* denote the specimen’s length, width, and thickness (mm), respectively.

#### 3.4.2. Experiment

**Figure 6:**
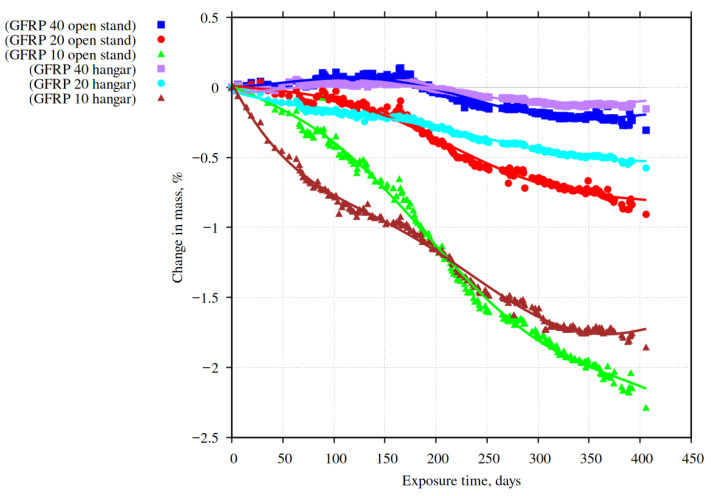
Mass change kinetics of GFRP samples during outdoor and hangar exposure.

**Figure 7:**
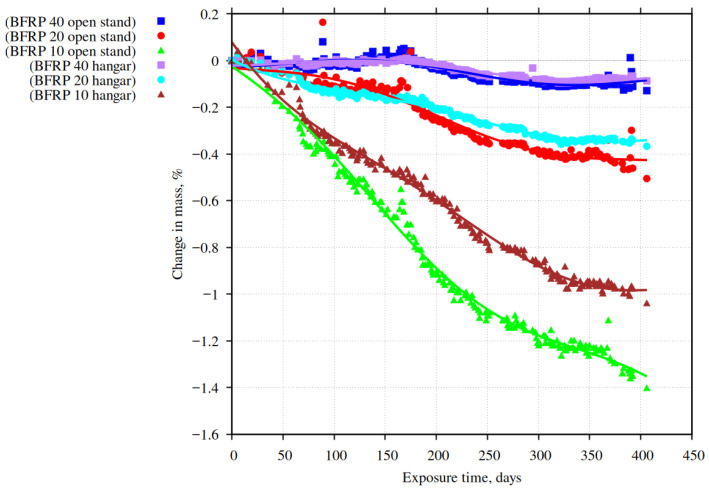
Mass change kinetics of BFRP samples during outdoor and hangar exposure.

A typical example of the kinetics of mass change for specimens of different sizes (10 × 10, 20 × 20, and 40 × 40 mm) over 405 days of exposure in an open stand and in a hangar are shown in Figure 6 and Figure 7. A general trend is observed—mass loss increases with exposure time (in agreement with [25,26,27,28]).

**Figure 8:**
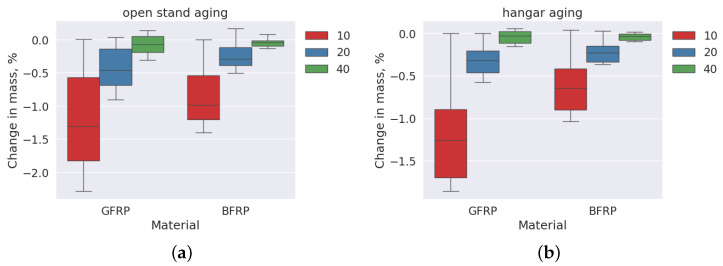
Effect of exposure conditions and sample size on mass changes represented as box-plots: (**a**) outdoor exposure, (**b**) hangar exposure.

The Figure 8 shows the relative mass changes after 405 days of exposure in an open site and in a hangar for GFRP and BFRP specimens with sizes of 10 × 10, 20 × 20, and 40 × 40 mm (four specimens for each size), presented as box plots.

Mass loss was highest in 10 × 10 mm samples during outdoor exposure (mean values: 2.3% for GFRP, 1.3% for BFRP) and decreased with sample size. In the hangar, where UV radiation was absent, mass loss decreased by 30–50%, indicating a significant role of internal thermal stresses in degradation. Seasonal mass gain (0.1–0.15%) in larger samples is attributed to moisture sorption, in agreement with previous works [25,26,28]. BFRP exhibits higher resistance to degradation than GFRP under both open-stand and hangar exposure conditions.

#### 3.4.3. Model Parameters

The parameters of the model (Equation (4)) for GFRP and BFRP, obtained by fitting the experimental data using Python 3.12.3, and averaged over four replicates for each specimen size, are presented in Table 7 and Table 8. The coefficients of determination $R^{2}$ are close to unity in all cases, indicating the high adequacy of the proposed model ($R^{2}=1$ would mean that all observed data perfectly match the model predictions).

The removal of degradation products from the bulk of GFRP and BFRP specimens during exposure on the open stand and in the hangar follows the law of Fickian desorption of low-molecular-weight products. This is supported by the pronounced dependence of the diffusion coefficient on the specimen dimensions, ranging from $1\times 10^{-4}$ mm^2^/day to 0.29 mm^2^/day.

The removal of degradation products from the specimen bulk is accompanied by moisture uptake, which varies with the season of exposure. The average moisture content over the exposure period ranges from 0.01% to 0.19%, depending on the material composition, specimen size, and exposure conditions.

The proposed diffusion-based degradation model Equation (4) was quantitatively validated due to the significant mass changes observed in small-size specimens. In typical exposure studies of polymer composite materials, considerably larger plates are employed [18,19,20,22,24,25]. The weighing sensitivity for such macro-specimens is insufficient to reliably detect their minor mass losses.

### 3.5. Dynamic Mechanical Analysis of GFRP and BFRP

Dynamic mechanical analysis (DMA) has been widely applied to identify dominant physicochemical transformations in polymer matrices of composite materials [18,32,41,44]. In the present study, DMA was employed to assess changes in the thermomechanical properties of GFRP and BFRP after long-term climatic aging.

**Figure 9:**
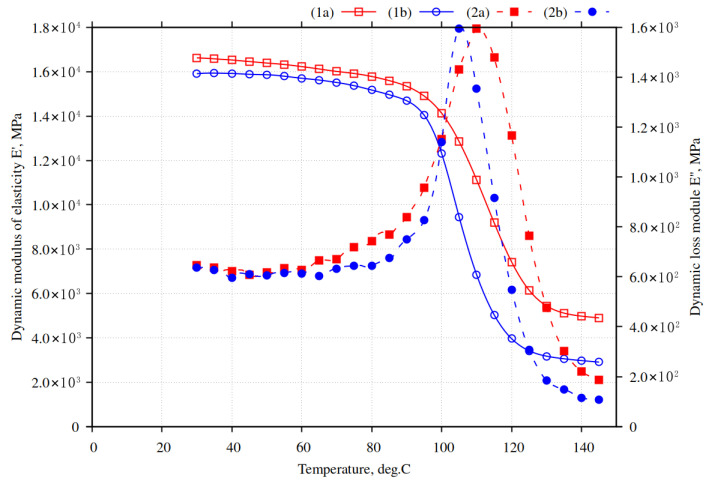
Temperature dependences of the storage modulus $E^{\prime }\left(T\right)$ (1) and the loss modulus $E^{\prime \prime }\left(T\right)$ (2) for the sun-facing side of GFRP in the initial state (a) and after 3 years of open-stand exposure in Yakutsk (b).

**Figure 10:**
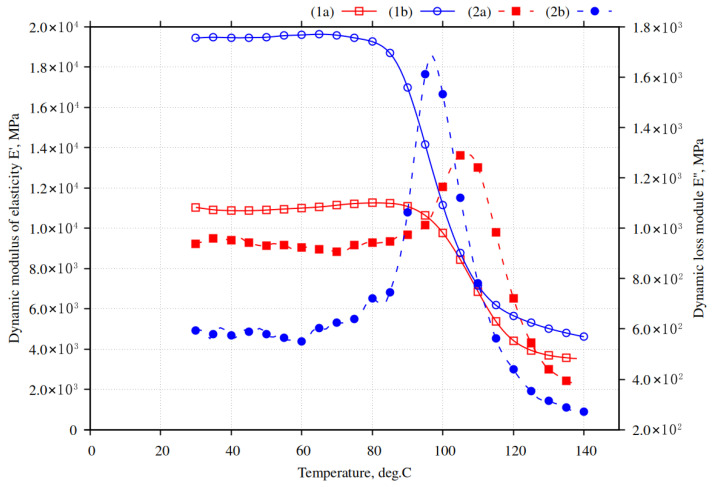
Temperature dependences of the storage modulus $E^{\prime }\left(T\right)$ (1) and the loss modulus $E^{\prime \prime }\left(T\right)$ (2) for the sun-facing side of BFRP in the initial state (a) and after 3 years of open-stand exposure in Yakutsk (b).

Figure 9 and Figure 10 present a typical example of the temperature dependences of the storage modulus $E^{\prime }\left(T\right)$ and the loss modulus $E^{\prime \prime }\left(T\right)$ for specimens cut from the sun-facing surfaces of GFRP and BFRP laminated plates in the as-received condition and after three years of open-stand exposure in Yakutsk. Table 9 and Table 10 presents the quantitative DMA results, for specimens cut from the sun-facing and shaded surfaces of GFRP and BFRP laminated plates in the as-received condition and after three years of open-stand exposure in Yakutsk.

For GFRP in the initial state, the onset and completion temperatures of the glass-to-rubber transition of the epoxy matrix were $T_{g1}=95$ °C and $T_{g2}=120$ °C, respectively, while the glass transition temperature determined from the $E^{\prime \prime }\left(T\right)$ peak was $T_{g}=107$ °C. After three years of environmental exposure, these temperatures decreased by 11–14 °C (Table 9), indicating epoxy matrix degradation due to solar UV radiation and internal thermal stresses. The ratio $E_{145}^{\prime }/E_{RT}^{\prime }$ decreased from 0.30±0.02 to 0.18±0.02 (a 40% reduction), consistent with the kinetic theory of high elasticity [45], reflecting reduced crosslink density and enhanced segmental mobility of macromolecular chains.

For BFRP, the initial characteristic temperatures $T_{g1}$, $T_{g}$, and $T_{g2}$ were 100±2 °C, 112±2 °C, and 126±1 °C, respectively. After three years of aging, these parameters decreased by only 4–6 °C (Table 10), and $E_{145}^{\prime }/E_{RT}^{\prime }$ dropped by 29%. These findings indicate less pronounced epoxy matrix degradation in BFRP compared to GFRP.

The comparative analysis (Table 11) demonstrates that the decrease in the glass transition temperatures ($T_{g1}$, $T_{g}$, and $T_{g2}$) and in the modulus ratio $E_{145}^{\prime }/E_{RT}^{\prime }$ is significantly smaller in BFRP than in GFRP after the same period of climatic aging. This indicates a higher resistance of the basalt–fiber-reinforced epoxy matrix to UV-induced and thermally induced degradation processes.

### 3.6. Thermomechanical Analysis of GFRP and BFRP Under Climatic Exposure

**Figure 11:**
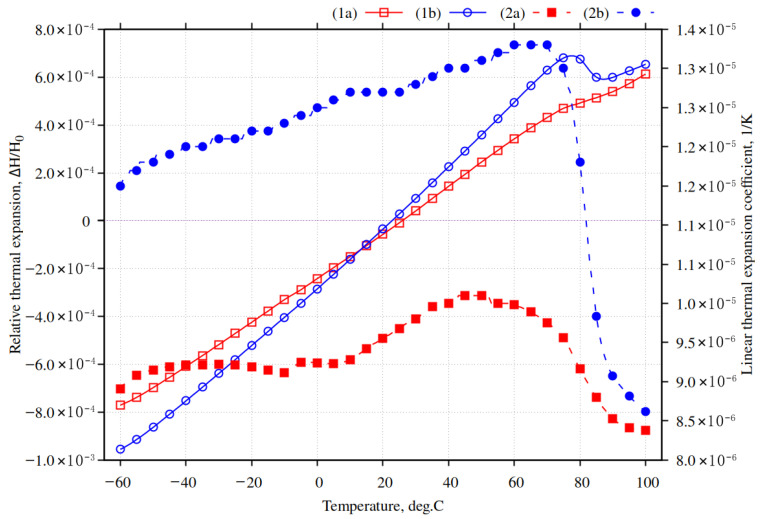
A typical example of the temperature dependences of the relative thermal expansion $\Delta H/H_{0}$ (1) and the coefficient of linear thermal expansion α (2) of GFRP in its initial state (a) and after 3 years of exposure at an outdoor stand in Yakutsk (b).

**Figure 12:**
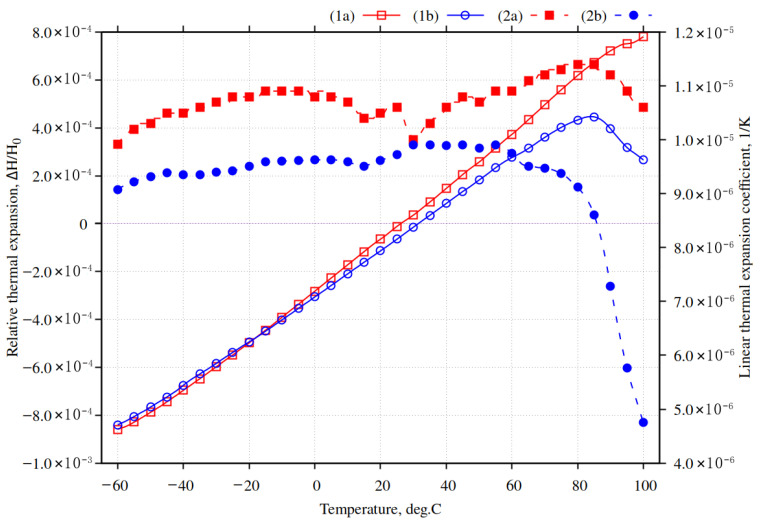
A typical example of the temperature dependences of the relative thermal expansion $\Delta H/H_{0}$ (1) and the coefficient of linear thermal expansion α (2) of BFRP in its initial state (a) and after 3 years of exposure at an outdoor stand in Yakutsk (b).

The thermomechanical analysis (TMA) conducted in this study provided additional insight into the aging mechanisms of GFRP and BFRP (Figure 11 and Figure 12, Table 12).

As shown in Figure 11, the relative thermal expansion $\Delta H/H_{0}$ of GFRP increases quasi-linearly over the temperature range from −60 °C to 80 °C in both the initial state and after 3 years of exposure to the climate of Yakutsk. In the entire glassy state of the epoxy matrix, the coefficient of linear thermal expansion α of GFRP increased by 20–30% after exposure. This increase can be explained by structural changes in the polymer matrix: prolonged exposure to solar UV radiation and thermal cycling induces partial degradation of the epoxy network, breaking some covalent and hydrogen bonds, and causes microcracking and loosening of the matrix. As a result, the intermolecular interactions that normally constrain the thermal motion of polymer chains are weakened, leading to higher segmental mobility. Additionally, internal thermal stresses accumulated during repeated heating and cooling cycles further disrupt the matrix–fiber interface, reducing the constraining effect of the glass fibers on the matrix. Together, these mechanisms result in an overall increase in the linear thermal expansion coefficient of the composite.

BFRP proved to be a more stable material. As shown in Figure 12 and Table 12, the coefficient of linear thermal expansion in the glassy state of BFRP decreased by 5–10% after 3 years of exposure. This slight reduction can be attributed to the higher thermal stability and lower intrinsic thermal expansion of basalt fibers, which more effectively constrain the matrix compared to glass fibers. Additionally, partial post-curing and relaxation of the epoxy matrix during long-term UV and thermal exposure can lead to local densification of the polymer network, reducing segmental mobility. The combination of a stiffer fiber–matrix interface and a denser matrix results in a minor overall decrease in the linear thermal expansion of the composite.

Overall, the analysis of the experimental results obtained by DMA and TMA confirms the occurrence of epoxy matrix degradation in the studied composites and demonstrates the superior climatic resistance of BFRP compared to GFRP.

## 4. Conclusions

Exposure of GFRP and BFRP laminates for three years under the Arctic climate of Yakutsk resulted in degradation of the epoxy matrix. This was confirmed by reductions in tensile strength and elastic modulus, decreased polymer content, lowered glass transition temperature, and altered coefficients of linear thermal expansion over a wide temperature range.

In studies of the environmental durability of polymer binders and other polymer composite materials, it is crucial not only to assess conventional mechanical properties but also to carefully monitor changes in the mass of the tested samples, identifying increases due to moisture sorption and decreases due to material degradation. Such measurements provide valuable insights into the predominance of moisture uptake versus structural deterioration.

Comparison of experimental results and mathematical modeling demonstrated that the primary cause of polymer matrix degradation is the action of abrupt internal stresses arising during thermal cycling under extreme cold climate conditions, due to differences in the coefficients of linear thermal expansion of glass and basalt fibers and the epoxy matrix. The proposed diffusion-based degradation model was validated by significant mass changes in small-sized samples during both outdoor and controlled hangar exposure.

Comparative analysis of mechanical properties, mass loss, dynamic mechanical and thermomechanical behavior, and matrix content consistently demonstrated the superior climatic durability of BFRP relative to GFRP. The practical implications of these findings support the informed selection and application of polymer composites, particularly BFRP, in construction materials for cold-region infrastructures. 

## Figures and Tables

**Table 1:** Physical and mechanical properties of the fabric grades.

Fabric Grade	BT-11 (100)	TR-560-30A (100)
Areal density, g/m^2^	351	560 (±40)
Thickness, mm	0.33 (±0.03)	0.45 (±0.05)
Tensile strength, N–warp	2610	2800
Tensile strength, N–weft	2120	2800
Warp yarn density, threads/cm	18	30 (±1)
Weft yarn density, threads/cm	8	30 (±1)
Weave type	twill 5/3	plain

**Table 2:** Number of transitions through temperature points in GFRP and BFRP during the 2023–2024.

Composite	0 °C	−18 °C	−33 °C
GFRP	438	262	163
BFRP	468	287	163

**Table 3:** Mechanical properties of GFRP and BFRP before and after exposure.

Composite	Property	Initial	After 1 Year	Rel. Change	After 2 Years	Rel. Change	After 3 Years	Rel. Change
GFRP	$\sigma_{t}$, MPa	611(±5)	542(±6)	0.89	465(±12)	0.76	475(±7)	0.78
	$E_{t}$, GPa	27.6(±0.1)	21.8(±0.4)	0.79	18.6(±0.4)	0.68	18.6(±0.4)	0.68
BFRP	$\sigma_{t}$, MPa	521(±3)	493(±15)	0.95	457(±5)	0.88	488(±6)	0.94
	$E_{t}$, GPa	19.1(±0.2)	18.9(±0.2)	0.99	16.4(±0.4)	0.88	16.4(±0.4)	0.86

**Table 4:** Binder mass fraction (wt%) in GFRP and BFRP before and after exposure.

Composite	Initial	After 1 Year	After 2 Years	After 3 Years
GFRP	24.8 (±0.4)	22.4 (±0.4)	20.0 (±0.4)	19.4 (±0.4)
BFRP	25.8 (±0.4)	25.3 (±0.4)	24.8 (±0.4)	24.3 (±0.4)

**Table 5:** Relative binder mass fraction (wt%) in GFRP and BFRP after exposure.

Composite	After 1 Year	After 2 Years	After 3 Years
GFRP	88 (±1)	76 (±1)	73 (±1)
BFRP	97 (±1)	94 (±1)	92 (±1)

**Table 6:** Surface average roughness parameter $R_{a}$ Equation (2) of GFRP, BFRP in the initial state and after 2 and 3 years of open-stand exposure in Yakutsk.

Composite	Initial (Sun-Facing/Shaded)	2 Years (Sun-Facing/Shaded)	3 Years (Sun-Facing/Shaded)
GFRP	1.61/5.61	5.92/5.62	5.86/3.46
BFRP	5.57/11.2	5.96/11.34	11.73/9.31

**Table 7:** Model parameters (Equation (4)) for GFRP.

Location	Open Stand			Hangar		
Size (mm)	10 × 10	20 × 20	40 × 40	10 × 10	20 × 20	40 × 40
$w_{s0}$ (%)	0.037	0.054	0.068	0.1900	0.0096	0.0018
$w_{s1}$ (%)	0.150	0.085	0.084	0.1700	0.0370	0.0570
φ (rad)	$-7.0\times 10^{5}$	$1.1\times 10^{6}$	$4.70\times 10^{5}$	$-2.5\times 10^{5}$	$4.7\times 10^{5}$	$-1.6\times 10^{3}$
$w_{d0}$ (%)	21	17	1.5	1.9	0.72	1.1
*D* (mm^2^/day)	0.0003	0.0001	0.0004	0.0078	0.0031	0.0002
$R^{2}$	0.994	0.983	0.946	0.993	0.988	0.955

**Table 8:** Model parameters (Equation (4)) for BFRP.

Location	Open Stand			Hangar		
Size (mm)	10 × 10	20 × 20	40 × 40	10 × 10	20 × 20	40 × 40
$w_{s0}$ (%)	0.0075	0.050	0.033	0.1400	0.0380	0.0018
$w_{s1}$ (%)	0.0840	0.0850	0.0360	0.0700	0.0380	0.0260
φ (rad)	$8.9\times 10^{5}$	$-1.1\times 10^{6}$	$-4.30\times 10^{5}$	$2.2\times 10^{5}$	$5.8\times 10^{4}$	$-4.5\times 10^{3}$
$w_{d0}$ (%)	3.7	2.3	0.15	1.2	0.39	0.25
*D* (mm^2^/day)	0.29	0.29	0.081	0.12	0.030	0.017
$R^{2}$	0.989	0.927	0.810	0.992	0.984	0.932

**Table 9:** DMA characteristics of GFRP in the initial state and after 2 and 3 years of open-stand exposure in Yakutsk.

Parameter	Initial (Sun-Facing/Shaded)	2 Years (Sun-Facing/Shaded)	3 Years (Sun-Facing/Shaded)
$E_{RT}^{\prime }$, GPa	11/15	15/15	19/16
$E_{145}^{\prime }/E_{RT}^{\prime }$	0.32/0.28	0.22/0.22	0.20/0.16
$T_{g1}$,°C	95/98	90/89	86/85
$T_{g}$,°C	109/109	99/97	95/95
$T_{g}$ *,°C	107/109	101/100	97/95
$T_{g2}$, °C	120/123	110/111	108/109

* Glass transition temperature from $E^{\prime \prime }$ peak. Notes: Measurement uncertainties are ±7% for $E_{RT}^{\prime }$ and $E_{145}^{\prime }$, and ±1 °C for $T_{g}$, $T_{g1}$, $T_{g}$*, $T_{g2}$.

**Table 10:** DMA characteristics of BFRP in the initial state and after 2 and 3 years of open-stand exposure in Yakutsk.

Parameter	Initial (Sun-Facing/Shaded)	2 Years (Sun-Facing/Shaded)	3 Years (Sun-Facing/Shaded)
$E_{RT}^{\prime }$, GPa	17/15	14/14	16/13
$E_{145}^{\prime }/E_{RT}^{\prime }$	0.30/0.27	0.24/0.25	0.19/0.21
$T_{g1}$,°C	98/102	99/99	96/96
$T_{g}$,°C	113/114	109/109	104/105
$T_{g}$ *, °C	111/112	110/110	106/106
$T_{g2}$,°C	126/126	120/120	115/115

* Glass transition temperature from $E^{\prime \prime }$ peak. Notes: Measurement uncertainties are ±7% for $E_{RT}^{\prime }$ and $E_{145}^{\prime }$, and ±1 °C for $T_{g}$, $T_{g1}$, $T_{g}$*, $T_{g2}$.

**Table 11:** Relative changes in DMA parameters of GFRP and BFRP after 3 years of open-stand exposure in Yakutsk.

Parameter Change	GFRP	BFRP
$\Delta T_{g1}$,°C	−11 to −13	−4 to −6
$\Delta T_{g}$,°C	−12 to −14	−5 to −6
$\Delta T_{g2}$,°C	−11 to −13	−5 to −6
$E_{145}^{\prime }/E_{RT}^{\prime }$ change	−40(±3)%	−29(±2)%

**Table 12:** Coefficients of linear thermal expansion α of GFRP and BFRP in the initial state and after 2 and 3 years of exposure at an outdoor stand in Yakutsk ($\alpha \cdot 10^{-6}$ 1/°C).

Temperature	GFRP			BFRP		
°C	Initial	2 Year	3 Year	Initial	2 Year	3 Year
−60	9.0	11.1	11.5	10.0	11.1	9.1
−20	9.2	11.7	12.1	10.5	11.9	9.4
0	9.3	11.8	12.5	10.2	12.0	9.6
20	9.6	12.3	12.8	10.3	12.1	9.6
60	10.0	12.2	13.2	10.5	12.3	9.4

Notes: Measurement uncertainties are $\pm 2\cdot 10^{-6}$ 1/°C.

## Data Availability

The original contributions presented in this study are included in the article. Further inquiries can be directed to the corresponding authors.

## Abbreviations

The following abbreviations are used in this manuscript:

ASTM	American Society for Testing and Materials
BFRP	Basalt Fiber Reinforced Polymer
CLTE	Coefficients of Linear Thermal Expansion
DMA	Dynamic Mechanical Analysis
GFRP	Glass Fiber Reinforced Polymer
ISO	International Organization for Standardization
T_*g*_	Glass Transition Temperature
TMA	Thermomechanical Analysis
UV	Ultraviolet
